# Supplementary material for: Matching based on biological categories in Orangutans (Pongo abelii) and a Gorilla (Gorilla gorilla gorilla)
Source: PeerJ. 2013 Sep 10;1:e158. doi: 10.7717/peerj.158 (PMC3775627; doi:10.7717/peerj.158)
Supplement: Supplemental Information 1 [file peerj-01-158-s001.docx]

Appendix A: Photographs used in Experiment 1.

| Species | Description | # Individuals  Depicted | Size (% of  Photo) | Background | Age | Body Depicted |
| --- | --- | --- | --- | --- | --- | --- |
|  |  |  |  |  |  |  |
| Golden Lion Tamarin | Very dark orange | 1 | 50 | Leaves | Adult | Whole Body |
|  | Orange | 1 | 50 | Blue, wood | Adult | Whole Body |
|  | Orange close-up | 1 | 75 | Green | Adult | Part Body |
|  | Dark orange | 1 | 75 | Watery Blue | Adult | Whole Body |
| Gibbon | Close-up dark-handed Gibbon | 1 | 100 | None | Adult | Face |
|  | Brown white-handed Gibbon | 1 | 75 | Tree and leaves | Adult | Part Body |
|  | Brown white-handed hanging | 1 | 50 | Tree and leaves | Adult | Whole Body |
|  | Dark Brown Mueller’s Gibbon | 1 | 75 | Tree and leaves | Adult | Part Body |
| Japanese Macaque | Brown and white | 1 | 50 | Water | Adult | Part Body |
|  | Brown and white | 3 | 75 | Snow | Adult | Part Body |
|  | Brown | 1 | 75 | Snow | Young | Whole Body |
|  | Brown and red close-up | 1 | 100 | White | Adult | Face |
| Proboscis | Golden and white close-up | 1 | 50 | Tree and leaves | Adult | Whole Body |
|  | Golden and white | 1 | 30 | Tree and leaves | Adult | Whole Body |
|  | Golden and white | 1 | 30 | Tree and leaves | Adult | Whole Body |
|  | Golden and white | 1 | 50 | Leaves | Adult | Whole Body |
| Lemur | Black and white | 1 | 50 | Grass | Adult | Whole Body |
|  | Grey, black, white | 3 | 75 | Tree and leaves | Adult | Part Body |
|  | Grey and white | 1 | 50 | Leaves | Young | Part Body |
|  | Grey, black, white | 1 | 50 | Red sand | Adult | Whole Body |

Appendix B: Photographs used in Experiment 2.

| Taxa | Species/  Genus | # Individuals  Depicted | Color | Size (% of  Photo) | Background | Body Depicted |  |
| --- | --- | --- | --- | --- | --- | --- | --- |
|  |  |  |  |  |  |  | |
| Insect | Spider | 1 | Black | 50 | Light | Full | |
|  | Butterflies | 2 | Mixed | 50 | Yellow | Full | |
|  | Bullworm | 1 | Light brown | 30 | Red | Full | |
|  | Flies | 3 | black | 25 | Dead snake | Full | |
|  | Alderfly | 1 | Brown, black | 25 | Leaf | Full | |
|  | Beetle | 1 | Dark brown | 50 | Green leaf | Full | |
| Reptile | Anaconda | 1 | Black, brown | 75 | Dirt | Full | |
|  | Snakehead | 1 | Mixed | 75 | Fuzzy | Face | |
|  | Water Lizard | 1 | Grey | 50 | Rocks | Full | |
|  | Alligator | 2 | Grey | 75 | Branches | Part | |
|  | Iguana | 1 | Pink, grey | 75 | Rocks | Part | |
|  | tortoise | 1 | Black | 50 | grass | Full | |
| Fish | Angelfish | 1 | Mixed | 75 | Coral | Full | |
|  | Pike | 2 | Silver | 25 | Plants | Full | |
|  | Blenny | 1 | Yellow, black | 75 | Fuzzy | Face | |
|  | Unknown | 1 | Black, silver | 50 | Water | Full | |
|  | Butterfly fish | 1 | Black, white | 75 | Blue | Full | |
|  | Stingray | 1 | Brown | 25 | rocks | Full | |
| Bird | Puffin | 1 | Black | 50 | Sky, water | Full | |
|  | Budgie | 1 | Yellow, black | 75 | Blue, green | Full | |
|  | Emus | 3 | Grey | 30 | Grass, dirt | Full | |
|  | Blackbird | 1 | Black | 50 | Berries | Full | |
|  | Owlet | 1 | Brown | 100 | Brown | Face | |
|  | Finch | 1 | Mixed | 50 | Grey | Full | |
| Mammal | Bull elephant seal | 1 | Brown | 75 | Rocks | Face | |
|  | Vole | 1 | Brown | 75 | Flowers | Full | |
|  | Armadillo | 1 | Brown | 50 | Sand | Full | |
|  | Black leopard | 1 | Black | 75 | Tree | Part | |
|  | Blackbucks | 2 | Brown, white | 50 | Dirt | Full | |
|  | Wolf pup | 1 | Grey | 50 | grass | Full | |
